# Influenza vaccine hesitancy and its influencing factors among community-dwelling older adults in Shihezi City, Xinjiang, Western China

**DOI:** 10.3389/fpubh.2026.1785390

**Published:** 2026-06-01

**Authors:** Xinmeng Wang, Shanshan Li, Xiaoju Li, Yiyao Li, Ruiying Peng, Yuhan Zhang

**Affiliations:** School of Medicine, Shihezi University, Shihezi, China

**Keywords:** influencing factors, influenza vaccine, older adults, vaccination behavior, vaccine hesitancy

## Abstract

**Objective:**

This study aims to examine the prevalence of influenza vaccine hesitancy and its associated factors among community-dwelling older adults in Shihezi, Xinjiang, thereby informing evidence-based strategies to enhance influenza vaccine uptake in older adults.

**Methods:**

A multi-stage cluster random sampling method was adopted to conduct a questionnaire survey among 1,540 community-dwelling older adults in Shihezi City, Xinjiang, in July 2023. Chi-square tests, univariate analysis, and multivariate logistic regression analysis were performed to examine the factors associated with influenza vaccine hesitancy among older adults.

**Results:**

Among the 1,474 older adults who completed the survey with valid responses, the prevalence of influenza vaccine hesitancy was 54.5% (804/1474). Univariate analysis showed that age, marital status, chronic diseases, self-rated health, knowledge of influenza and influenza vaccines, perceptions of vaccine efficacy and safety, recommendations from healthcare providers, prior vaccination history, and certain subscale scores of the 5C scale were significantly associated with vaccine hesitancy (*p* < 0.05). Multivariate analysis revealed that complacency was an independent risk factor for vaccine hesitancy (OR = 3.204, 95% CI: 2.439–4.208, *p* < 0.001), while confidence, recognition of vaccine efficacy and safety, and moderate to high levels of knowledge about influenza and vaccines were protective factors (*p* < 0.05).

**Conclusion:**

Influenza vaccine hesitancy among community-dwelling older adults in Shihezi, Xinjiang, is affected by multiple factors. To reduce hesitancy and increase vaccination coverage, targeted education on influenza and vaccines should be implemented to improve disease risk awareness and vaccine confidence, along with enhanced vaccination recommendations from healthcare providers.

## 1 Introduction

Influenza, or influenza, is an acute respiratory infectious disease caused by influenza virus. It mainly occurs in winter and spring, posing a serious threat to human health and causing a high burden of disease. Compared with other age groups (1), older adults are more likely to have severe complications and a higher risk of death after influenza due to weakened immunity. In particular, older adults with multiple chronic diseases at the same time or in succession are at high risk of influenza virus infection (2). Influenza infection in older adults can pose a health threat to older adults with chronic diseases. The symptoms after infection are more serious and the risk of death is increased. 65–96% of deaths caused by influenza occur in older adults aged 65 years and older (3). Currently, Influenza vaccine is still the most effective way to prevent influenza in older adults. The Influenza vaccine vaccination can produce significant economic benefits and improve the health utility of older adults, thereby reducing the influenza prevalence and disease burden in older adults.

Influenza vaccination has been proven to be the most effective and cost-effective public health intervention to prevent influenza and its complications (4). WHO recommends Influenza vaccine vaccination for health workers and older adults as the highest priority (5). In Canada, Belgium, the Netherlands, Uruguay, and the United States, the Influenza vaccine coverage rate of chronic disease patients was over 40%. In South Korea, the Influenza vaccine coverage rate of chronic disease patients was 56.68% (6). In some areas of China, Influenza vaccine vaccination has been implemented free of charge for people aged 60 and above. However, the Influenza vaccine vaccination rate is still low, only about 3% (7), which is much lower than the vaccination rate of older adults in the United States (8), Japan (9), Denmark (10), Portugal, Spain and the Netherlands (11). Wang Conglin et al. (12) used a cross-sectional study to investigate the Influenza vaccine coverage rate of 1,265 older adults people aged 60 years and above in rural areas of Tianjin, China, and the results showed that the Influenza vaccine coverage rate was 7.4%. Sun Minying et al. (13) used a multi-stage stratified random sampling method to investigate the Influenza vaccine coverage rate of older adults in some communities in Guangzhou, and the results showed that the influenza vaccination rate was 19.59%. The Influenza vaccine coverage rate was still low in older adults in China. Studies have shown that the average cost of hospitalization due to influenza infection in older adults is twice that of other adults, and the average cost per illness in older adults aged ≥60 years is much higher than that in older adults aged < 60 year. Influenza vaccination can effectively reduce the disease burden of influenza, and older adults with multimorbidity can significantly reduce their hospitalization and mortality burden by receiving the influenza vaccination (14).

Although the coverage of influenza vaccination is gradually improving worldwide, influenza vaccine hesitancy is common, which is affecting the effectiveness of influenza vaccination. Vaccine hesitancy refers to a continuous process between vaccination intention and actual vaccination behavior. Under the situation of vaccine services being offered, vaccine recipients delay or refuse to receive the vaccine, which is the main cause of insufficient vaccination rate and coverage. The World Health Organization (WHO) listed vaccine hesitancy as one of the 10 threats to global health in 2019, and proposed that the success of global immunization campaigns depends on achieving and maintaining high vaccination rates (15). Therefore, as one of the important factors hindering the vaccination rate of older adults, “vaccine hesitancy” caused by complacency, convenience and trust should be paid attention to and addressed as a priority of national immunization programs.

Current research and practice indicate that influenza vaccine hesitancy remains at a relatively high level across populations, particularly among older adults (16). Many older adults adopt a wait-and-see attitude or refuse vaccination due to insufficient confidence in vaccine safety, concerns about potential adverse reactions (17), or complacency arising from the belief that they are healthy and at low risk of influenza infection (18). This widespread hesitancy has become a key barrier to achieving adequate influenza vaccination coverage and establishing effective herd immunity. Vaccine hesitancy is a complex phenomenon shaped by the interaction of multiple factors, including individual psychology, social environment, and vaccine services. To systematically explain its psychological antecedents, Betsch et al. proposed the 5C model, which encompasses five core dimensions: Confidence, Complacency, Constraints, Calculation, and Collective Responsibility. Among older adults, each dimension carries specific practical implications. Confidence concerns trust in vaccine safety and efficacy; Complacency involves underestimation of influenza risk; Constraints reflect practical barriers such as limited mobility and restricted access to information; Calculation emphasizes the ability to weigh vaccine benefits against risks; and Collective Responsibility reflects older adults’ sense of duty to protect their families and communities through vaccination. Therefore, using the 5C model to analyze influenza vaccine hesitancy among older adults in Shihezi City, Xinjiang, can contribute to a comprehensive understanding of the underlying psychological mechanisms and provide a foundation for developing targeted intervention strategies.

In this study, a cross-sectional survey was conducted on the vaccination status of older adults in Shihezi City in July 2023. This study aims to examine influenza vaccination coverage and vaccine hesitancy among older adults in Shihezi City, identify factors associated with vaccine hesitancy, and thereby promote vaccination uptake in this population, ultimately providing a scientific and theoretical basis for developing and implementing targeted interventions.

## 2 Objects and methods

### 2.1 Study setting and participants

Community-dwelling older adults in Shihezi City, Xinjiang, were recruited as study participants. Eligibility criteria included: (1) residence in the surveyed community for >6 months; (2) ability to understand and read Chinese and to answer questions normally; and (3) provision of informed consent and voluntary participation. Older adults with cognitive impairment, hearing deficits, or language disorders were excluded.

This study conducted a questionnaire survey among 1,540 community-dwelling older adults in Shihezi City, Xinjiang, in July 2023, using a multi-stage cluster random sampling method. The sampling procedure involved three stages. First, five subdistricts with community neighborhood committees were selected according to the geographical distribution of Shihezi City. Second, one community was randomly selected from each subdistrict using simple random sampling, yielding five communities in total. Third, 300 older adults were randomly selected from each community based on the rosters provided by the respective community health service stations.

A total of 1,540 participants were recruited and invited to complete the questionnaire in this survey. After collection, 20 invalid questionnaires with incomplete core items and patterned responses were excluded via preliminary screening, leaving 1,520 valid questionnaires. To guarantee the accuracy and reliability of data analysis, another 46 questionnaires with missing key variables related to vaccine hesitancy were further excluded. Ultimately, 1,474 participants were included in the final statistical analysis. This survey on the current status of vaccine hesitancy was approved by the Ethics Committee of the First Affiliated Hospital of Shihezi University School of Medicine (Approval No. KJ2021-135-01). All eligible participants provided written informed consent prior to data collection.

### 2.2 Sample size calculation

Sample size was calculated using the formula for cross-sectional studies: $n=\left(Z^{2}\times p\times \left(1-p\right)\right)÷d^{2}$ . Parameters were set as follows: 95% confidence level (Z = 1.96) and margin of error d = 0.05. Based on published literature reporting influenza vaccine hesitancy rates among older adults ranging from 33.5 to 69.7% (16, 17, 19), a conservative estimate of 33.5% was adopted for sample size calculation, which gave an initial sample of 343 individuals. After accounting for the design effect of multi-stage sampling and an anticipated non-response rate of 15%, the sample size was adjusted to a minimum of 789 participants.

### 2.3 Measures and variable definitions

In accordance with the World Health Organization’s definition and previous studies (20, 21), influenza vaccine hesitancy was assessed using a single question: “Based on your actual situation, please indicate your true intention regarding influenza vaccination.” The response options were: “definitely will get vaccinated,” “probably will get vaccinated,” “uncertain,” “probably will not get vaccinated,” and “definitely will not get vaccinated.” Respondents who answered “uncertain,” “probably will not get vaccinated,” or “definitely will not get vaccinated” were classified as having vaccine hesitancy.

The following core concepts were defined for this study: (1) Vaccine hesitancy – a psychological state characterized by delay or refusal of vaccination despite the availability of vaccine services. (2) Vaccination intention – an individual’s subjective attitude indicating willingness to receive vaccination. (3) Vaccination behavior – the actual act of receiving vaccination. In this study, vaccination behavior was assessed by asking participants whether they had received an influenza vaccine in the past 2 years.

General information questionnaire: including demographic data such as gender, age, marital status, education level; Health-related information, such as self-rated health status, medical insurance status, influenza knowledge score, influenza vaccine knowledge score, whether to consider the effectiveness and safety of vaccine, whether to have chronic diseases, influenza vaccination in the past 2 years, etc.

Influenza and influenza vaccine knowledge score: The knowledge assessment was adapted from items used in recent knowledge-attitude-practice (KAP) surveys on influenza vaccination (22, 23). The scale consisted of 14 items in total, with 7 items measuring influenza knowledge and 7 items measuring influenza vaccine knowledge. Each correct answer was assigned 1 point, whereas incorrect answers or “unsure” responses were assigned 0 points. The total score for each section ranged from 0 to 7, with higher scores reflecting better knowledge of influenza and influenza vaccines among older adults. Based on the score distribution, participants were classified into three groups: the low knowledge group (0–2 points), the moderate knowledge group (3–4 points), and the high knowledge group (5–7 points).

Vaccine Hesitancy 5C Scale: The 5C scale, originally developed by Betsch et al. (24), was administered using its culturally adapted and psychometrically validated Chinese version. In the present study, the Cronbach’s *α* coefficients for the 5C subscales ranged from 0.670 to 0.933 (25), demonstrating good internal consistency. The scale comprises 15 items (3 items per dimension), with each item rated on a 5-point Likert scale (1 = strongly disagree, 5 = strongly agree). The dimensions of Confidence, Calculation, and Collective Responsibility were positively scored, whereas Complacency and Constraints were reverse-scored. The total possible score ranged from 15 to 75, with higher scores reflecting more favorable attitudes toward vaccination. In this study, subscale scores were treated as continuous variables in the analysis.

### 2.4 Statistical analysis

Descriptive statistics were used to characterize the distribution of individual characteristics across scale scores. Categorical data were reported as frequencies and percentages (%), and continuous data as mean ± standard deviation (x̄ ± s). The chi-square test was applied to explore factors associated with influenza vaccine hesitancy. Independent-samples *t*-test and one-way ANOVA were performed to compare 5C subscale scores across different sociodemographic characteristics, prior vaccination history, and levels of vaccine hesitancy. Multivariable logistic regression analysis was used to explore the influencing factors of influenza vaccine hesitancy among community-dwelling older adults. The Hosmer–Lemeshow test was used to assess the goodness-of-fit of the logistic regression model. Multicollinearity was examined using the variance inflation factor (VIF), with a VIF threshold of <10 indicating no severe multicollinearity among independent variables. All statistical analyses were conducted using IBM SPSS Statistics version 26.0. A two-sided *p*-value < 0.05 was considered statistically significant.

## 3 Results

### 3.1 Basic demographic characteristics

A total of 1,474 older adults were enrolled in this study. Females accounted for 63.3% of the sample, and males for 36.7%. The predominant age group was 70–80 years (38.9%). Married individuals constituted 68.1% of the participants. The majority had an education level of junior high school or below (77.4%). Regarding occupation, workers (32.6%) and those employed in commercial or service industries (30.6%) were the two largest groups. Most participants reported a monthly income of 3,000–5,000 RMB (52.7%). Chronic diseases were present in 61.5% of the sample, and 49.3% rated their health as good. Nearly all participants (98.4%) had medical insurance, while only 8.1% had a medical background. With respect to knowledge scores, 75.8% of participants fell into the high-scoring group for influenza knowledge, and 62.7% for influenza vaccine knowledge. Regarding vaccine perceptions, 37.7% of older adults indicated that perceived vaccine effectiveness would influence their vaccination intention, and 33.2% indicated that perceived vaccine safety would influence their vaccination intention (see Table 1).

**Table 1:** Basic characteristics of the study subjects.

Characteristic	Category	*n* (%)
Gender	Male	541 (36.7)
	Female	933 (63.3)
Age	60–70	511 (34.7)
	70–80	573 (38.9)
	80–90	381 (25.8)
	90 and above	9 (0.6)
Marital status	Unmarried	7 (0.5)
	Married	1,004 (68.1)
	Other	463 (31.4)
Education level	Junior high school or below	1,141 (77.4)
	High school (vocational/technical secondary school)	199 (13.5)
	Other	134 (9.1)
Occupation	Government/public institution staff	83 (5.6)
	Commercial/service workers	451 (30.6)
	Professional and technical personnel	163 (11.1)
	Workers	480 (32.6)
	Farmers	241 (16.4)
	Other	56 (3.8)
Monthly income	<3,000	517 (35.1)
	3,000–5,000	777 (52.7)
	5,000–7,000	151 (10.2)
	≥7,000	29 (2.0)
Chronic disease	Yes	906(61.5)
	No	568 (38.5)
Self-rated health status	Good	726 (49.3)
	Fair	438 (29.7)
	Poor	310 (21.0)
Medical insurance coverage	Yes	1,450 (98.4)
	No	24 (1.6)
Medical background	Yes	120 (8.1)
	No	1,354 (91.9)
Influenza knowledge score	Low	50 (3.4)
	Medium	306 (20.8)
	High	1,118 (75.8)
Influenza vaccine knowledge score	Low	136 (9.2)
	Medium	414(28.1)
	High	924 (62.7)
Consider influenza vaccine effectiveness	Yes	555 (37.7)
	No	919 (62.3)
Consider influenza vaccine safety	Yes	489 (33.2)
	No	985 (66.8)
Recommendation from healthcare workers	Yes	255 (17.3)
	No	1,219 (82.7)
Influenza vaccination in 2020	Yes	395 (26.8)
	No	1,079 (73.2)
Influenza vaccination in 2021	Yes	460 (31.2)
	No	1,014 (68.8)

### 3.2 Univariate analysis of factors associated with influenza vaccine hesitancy among older adults

Univariate analysis revealed that the following factors were significantly associated with vaccine hesitancy among participants (*p* < 0.05): age group, marital status, presence of chronic diseases, self-rated health, influenza knowledge score, influenza vaccine knowledge score, perceptions of vaccine efficacy and safety, receipt of a healthcare provider’s vaccination recommendation, and influenza vaccination history in both 2021 and 2022. In contrast, no significant differences in vaccine hesitancy distribution were observed for gender, education level, occupation, monthly income, medical insurance coverage, or medical background (*p* > 0.05) (see Table 2). The coding scheme for variables is presented in Table 3.

**Table 2:** Comparison of vaccine hesitancy among study subjects with different characteristics.

Variable	Category	Vaccine Hesitancy		*χ*^2^ value	*P*-value
		No (%)	Yes (%)		
Gender	Male	235 (43.4)	306 (56.6)	1.402	0.236
	Female	435 (46.6)	498 (53.4)		
Age	60–70	223 (43.6)	288 (56.4)	8.447	0.038
	70–80	250 (43.6)	323 (56.4)		
	80–90	195 (51.2)	186 (48.8)		
	90 and above	2 (22.2)	7 (77.8)		
Marital status	Unmarried	2 (28.6)	5 (71.4)	6.330	0.042
	Married	478 (47.6)	526 (52.4)		
	Other	190 (41.0)	273 (59.0)		
Education level	Junior high school or below	512 (44.9)	629 (55.1)	0.815	0.665
	High school (vocational/technical secondary school)	96 (48.2)	103 (51.8)		
	Other	62 (46.3)	72 (53.7)		
Occupation	Government/public institution staff	45 (54.2)	38 (45.8)	4.891	0.429
	Commercial/service workers	194 (43.0)	257 (57.0)		
	Professional and technical personnel	74 (45.4)	89 (54.6)		
	Workers	222 (46.2)	258 (53.8)		
	Farmers	106 (44.0)	135 (56.0)		
	Other	29 (51.8)	27 (48.2)		
Monthly income	<3,000	216 (41.8)	301 (58.2)	6.186	0.103
	3,000–5,000	376 (48.4)	401 (51.6)		
	5,000–7,000	64 (42.4)	87 (57.6)		
	≥7,000	14 (48.3)	15 (51.7)		
Chronic disease	Yes	302 (33.3)	604 (66.7)	140.862	<0.001
	No	368 (64.8)	200 (35.2)		
Self-rated health status	Good	300 (41.3)	426 (58.7)	11.925	0.003
	Fair	207 (47.3)	231 (52.7)		
	Poor	163 (52.6)	147 (47.4)		
Medical insurance coverage	Yes	662 (45.7)	788 (54.3)	1.446	0.229
	No	8 (33.3)	16 (66.7)		
Medical background	Yes	47 (39.2)	73 (60.8)	2.083	0.149
	No	623 (46.0)	731 (54.0)		
Influenza Knowledge score	Low	15 (30.0)	35 (70.0)	6.582	0.037
	Medium	131 (42.8)	175(57.2)		
	High	524 (46.9)	594(53.1)		
Influenza vaccine knowledge score	Low	29 (21.3)	107 (78.7)	70.933	<0.001
	Medium	148 (35.7)	266 (64.3)		
	High	493 (53.4)	431 (46.6)		
Consider influenza vaccine effectiveness	Yes	211 (38.0)	344 (62.0)	19.855	<0.001
	No	459 (49.9)	460 (50.1)		
Consider influenza vaccine safety	Yes	181 (37.0)	308 (63.0)	21.025	<0.001
	No	489 (49.6)	496 (50.4)		
Recommendation from healthcare workers	Yes	235 (92.2)	20 (7.8)	271.253	<0.001
	No	435 (35.7)	784 (64.3)		
Influenza vaccination in 2020	Yes	365 (92.4)	30(7.6)	479.753	<0.001
	No	305 (28.3)	774 (71.7)		
Influenza vaccination in 2021	Yes	429 (93.3)	31 (6.7)	616.385	<0.001
	No	241 (23.8)	773 (76.2)		

**Table 3:** Assignment of relevant variables.

Variable	Assignment
Gender	1 = Male; 2 = Female
Age	1 = 60–70; 2 = 70–80; 3 = 80–90; 4 = 90+
Marital status	1 = Unmarried; 2 = Married; 3 = Other
Education level	1 = Middle school or below; 2 = High school (technical school/secondary specialized school); 3 = College or above
Occupation	1 = Government or public institution; 2 = Commercial or service industry; 3 = Professional and technical personnel; 4 = Worker; 5 = Farmer; 6 = Other
Monthly income	1 = <3,000; 2 = 3,000–5,000; 3 = 5,000–7,000; 4=≥7,000
Have chronic disease	0 = No; 1 = Yes
Self-rated health status	1 = Good; 2 = Fair; 3 = Poor
Health insurance coverage	0 = No; 1 = Yes
Medical background	0 = No; 1 = Yes
Influenza knowledge score	1 = Low; 2 = Medium; 3 = High
Influenza vaccine knowledge score	1 = Low; 2 = Medium; 3 = High
Consider influenza vaccine effectiveness	0 = No; 1 = Yes
Consider influenza vaccine safety	0 = No; 1 = Yes
Healthcare provider recommendation	0 = No; 1 = Yes
Received influenza vaccine in 2020	0 = No; 1 = Yes
Received influenza vaccine in 2021	0 = No; 1 = Yes
Vaccine hesitancy	0 = Not hesitant; 1 = Hesitant

### 3.3 Dimensional analysis of the 5C model for influenza vaccine hesitancy among community-dwelling older adults

Univariate analyses showed that age group was significantly associated with scores on the Complacency, Constraints, and Collective Responsibility dimensions (*p* < 0.05). Scores of the Confidence and Complacency dimensions differed significantly among older adults with different marital statuses (*p* < 0.05). Confidence, Complacency and Constraints dimension scores were found in older adults with varying monthly income levels (*p* < 0.05). The presence of chronic diseases was significantly associated with scores on the Confidence, Complacency, Constraints, and Collective Responsibility dimensions (all *p* < 0.001). Vaccine hesitancy status was significantly associated with scores on all five dimensions of the 5C scale (all p < 0.001). In contrast, no significant differences in any 5C dimension scores were observed for gender, education level, or occupation (*p* > 0.05) (see Table 4).

**Table 4:** Univariate analysis of each dimension of the 5C influenza vaccine hesitancy scale in older adults.

Item	*n*	Confidence x̄ ± s	t/*F*-value	*p*-value	Complacency x̄ ± s	t/F value	*P*-value	Calculation x̄ ± s	t/F value	*P*-value	Constraints x̄ ± s	t/F value	*p-*value	Collective responsibility x̄ ± s	t/F value	*p*-value
Gender			−0.336	0.737		0.631	0.528		1.740	0.082		−0.025	0.98		−0.500	0.617
Male	541	8.58 ± 4.001			9.80 ± 3.878			6.74 ± 0.741			9.54 ± 2.418			8.63 ± 2.787		
Female	933	8.66 ± 4.022			9.66 ± 3.927			6.67 ± 0.759			9.54 ± 2.482			8.70 ± 2.683		
Age			1.604	0.187		2.807	0.038		0.927	0.427		3.424	0.017		3.690	0.012
60–70	511	8.44 ± 3.979			9.96 ± 3.875			6.72 ± 0.736			9.40 ± 2.421			8.53 ± 2.695		
70–80	573	8.56 ± 3.953			9.80 ± 3.839			6.71 ± 0.758			9.45 ± 2.480			8.55 ± 2.788		
80–90	381	9.00 ± 4.149			9.23 ± 4.018			6.65 ± 0.776			9.87 ± 2.454			9.07 ± 2.637		
90 and above	9	7.89 ± 3.408			10.33 ± 4.243			6.89 ± 0.333			8.78 ± 2.279			8.22 ± 2.048		
Marital status			4.795	0.008		3.086	0.046		0.864	0.422		2.238	0.107		1.946	0.143
Unmarried	7	8.71 ± 3.546			10.29 ± 3.988			7.00 ± 0.000			8.71 ± 2.563			7.86 ± 2.545		
Married	1,044	8.85 ± 4.004			9.54 ± 3.961			6.71 ± 0.751			9.63 ± 2.461			8.77 ± 2.739		
Other	463	8.15 ± 4.007			10.08 ± 3.771			6.68 ± 0.764			9.36 ± 2.443			8.49 ± 2.679		
Education level			0.366	0.693		0.730	0.482		0.428	0.652		0.571	0.565		0.009	0.991
Junior high school or below	1,141	8.63 ± 3.976			9.77 ± 3.894			6.70 ± 0.757			9.50 ± 2.464			8.68 ± 2.715		
High school (vocational/technical secondary school)	199	8.79 ± 4.079			9.41 ± 4.080			6.67 ± 0.732			9.64 ± 2.429			8.66 ± 2.742		
Other	134	8.41 ± 4.245			9.66 ± 3.770			6.75 ± 0.753			9.70 ± 2.453			8.66 ± 2.767		
Occupation			0.626	0.680		0.765	0.575		0.221	0.953		1.356	0.238		0.169	0.974
Government/public institution staff	83	9.02 ± 4.291			9.07 ± 3.904			6.77 ± 0.704			9.96 ± 2.634			8.87 ± 2.726		
Commercial/service workers	451	8.41 ± 3.970			9.85 ± 3.906			6.68 ± 0.763			9.38 ± 2.468			8.62 ± 2.680		
Professional and technical personnel	163	8.60 ± 4.038			9.85 ± 3.917			6.71 ± 0.768			9.51 ± 2.458			8.64 ± 2.865		
Workers	480	8.80 ± 4.000			9.61 ± 3.919			6.71 ± 0.759			9.68 ± 2.394			8.73 ± 2.798		
Farmers	241	8.55 ± 3.944			9.85 ± 3.875			6.69 ± 0.740			9.40 ± 2.478			8.63 ± 2.527		
Other	56	8.80 ± 4.333			9.45 ± 3.995			6.68 ± 0.741			9.63 ± 2.519			8.64 ± 2.857		
Monthly income			2.848	0.036		4.191	0.006		0.434	0.728		6.492	<0.001		1.605	0.186
<3,000	517	8.25 ± 3.876			10.16 ± 3.755			6.70 ± 0.767			9.17 ± 2.487			8.48 ± 2.641		
3,000–5,000	777	8.91 ± 4.066			9.39 ± 3.968			6.70 ± 0.748			9.78 ± 2.421			8.80 ± 2.784		
5,000–7,000	151	8.53 ± 4.053			9.87 ± 3.972			6.75 ± 0.704			9.51 ± 2.400			8.62 ± 2.594		
≥7,000	29	8.62 ± 4.395			9.41 ± 3.978			6.59 ± 0.907			9.90 ± 2.526			9.07 ± 2.987		
Chronic disease			13.432	<0.001		−13.133	<0.001		−1.399	0.162		11.953	<0.001		10.884	<0.001
Yes	568	10.30 ± 3.838			8.11 ± 3.928			6.67 ± 0.730			10.46 ± 2.421			9.61 ± 2.705		
No	906	7.58 ± 3.759			10.71 ± 3.547			6.72 ± 0.767			8.96 ± 2.300			8.09 ± 2.563		
Vaccine hesitancy			49.578	<0.001		−70.230	<0.001		−7.286	<0.001		50.431	<0.001		43.659	<0.001
Not hesitant	670	12.11 ± 2.979			5.96 ± 2.689			6.55 ± 0.742			11.68 ± 1.659			10.91 ± 1.851		
Hesitant	804	5.73 ± 1.916			12.84 ± 0.644			6.83 ± 0.739			7.75 ± 1.330			6.81 ± 1.751		

### 3.4 Analysis on influencing factors of influenza vaccine hesitancy in older adults in community

All variables that were either significant in previous studies or in the univariate analysis were entered into the binary logistic regression model, including each dimension of the 5C scale (Confidence, Complacency, Constraints, Calculation, and Collective Responsibility), consideration of influenza vaccine efficacy and safety, influenza knowledge score, influenza vaccine knowledge score, self-rated health, presence of chronic diseases, healthcare provider recommendation for vaccination, influenza vaccination status in 2021 and 2022, age, marital status, monthly income, education level, and occupation. The Hosmer–Lemeshow test was used to evaluate the goodness-of-fit of the model, yielding a *χ*^2^ value of 6.676 (*p* = 0.572), suggesting that the model fit the data well. Multicollinearity among the independent variables was assessed using the variance inflation factor (VIF). The maximum VI*F* value was 7.435 (for Complacency), and all other VIF values were below 5, indicating that no severe multicollinearity was present; therefore, all variables were retained in the regression model for analysis.

The multivariate logistic regression analysis revealed that, among the 5C model dimensions, higher scores on the Confidence dimension were associated with a lower risk of influenza vaccine hesitancy among older adults (OR = 0.827, 95% CI: 0.823–0.723, *p* = 0.003), whereas higher scores on the Complacency dimension were associated with a higher risk of vaccine hesitancy (OR = 3.204, 95% CI: 2.439–4.208, *p* < 0.001). The dimensions of Constraints, Calculation, and Collective Responsibility showed no significant association with vaccine hesitancy (*p* > 0.05). Regarding vaccine perceptions, older adults who considered the influenza vaccine to be effective (OR = 0.048, 95% CI: 0.017–0.132, *p* < 0.001) or safe (OR = 0.054, 95% CI: 0.021–0.141, p < 0.001) had a significantly lower risk of vaccine hesitancy. In terms of knowledge level, compared with the low-scoring group for influenza knowledge, both the moderate-level group (OR = 0.095, 95% CI: 0.011–0.810, *p* = 0.031) and the high-level group (OR = 0.111, 95% CI: 0.014–0.905, *p* = 0.040) exhibited a reduced risk of vaccine hesitancy. For influenza vaccine knowledge, the high-level group also had a lower risk of vaccine hesitancy compared with the low-level group (OR = 0.286, 95% CI: 0.088–0.929, *p* = 0.037), while no statistically significant difference was observed between the moderate-level and low-level groups (*p* = 0.174). After controlling for other factors, no statistically significant differences were found for age, marital status, education level, monthly income, occupation, presence of chronic diseases, self-rated health, healthcare provider recommendation for vaccination, or influenza vaccination history in 2021 and 2022 (*p* > 0.05). Furthermore, the model tested the interaction between chronic disease status and vaccine safety (*p* > 0.05), the interaction between healthcare provider recommendation and prior vaccination history (*p* > 0.05), and the interaction between healthcare provider recommendation and vaccine knowledge score (*p* > 0.05); none of these interactions were statistically significant (see Table 5).

**Table 5:** Multivariate logistic regression analysis of influencing factors for influenza vaccine hesitancy among older adults.

Variable	*B* value	SE value	Wald *χ*^2^ value	*p*-value	OR value	95%CI
Confidence	−0.195	0.066	8.671	0.003	0.827	0.823–0.723
Complacency	1.164	0.139	70.083	<0.001	3.204	2.439–4.208
Constraints	−0.062	0.110	0.315	0.575	0.940	0.757–1.167
Calculation	0.044	0.186	0.056	0.813	1.045	0.726–1.505
Collective Responsibility	−0.117	0.087	1.795	0.180	0.890	0.750–1.056
Consider vaccine effectiveness (ref: no)	−3.040	0.517	34.626	<0.001	0.048	0.017–0.132
Consider vaccine safety (ref: no)	−2.922	0.492	35.302	<0.001	0.054	0.021–0.141
Influenza knowledge score (ref: low)						
Medium	−2.351	1.092	4.636	0.031	0.095	0.011–0.810
High	−2.197	1.070	4.218	0.040	0.111	0.014–0.905
Influenza vaccine knowledge score (ref: low)						
Medium	−0.835	0.614	1.850	0.174	0.434	0.130–1.445
High	−1.253	0.602	4.339	0.037	0.286	0.088–0.929
Self-rated health status (ref: good)						
Fair	−0.410	0.346	1.401	0.237	0.664	0.337–1.309
Poor	−0.839	0.443	3.590	0.058	0.432	0.181–1.029
Chronic disease (ref: no)	0.635	0.351	3.268	0.071	1.886	0.948–3.753
Healthcare provider recommendation (ref: no)	−1.040	0.657	2.510	0.113	0.353	0.098–1.280
Influenza vaccination in 2021(ref: no)	−0.978	0.558	3.080	0.079	0.376	0.126–1.121
Influenza vaccination in 2022(ref: no)	−0.666	0.494	1.814	0.178	0.514	0.195–1.354
Age (ref: 60–70)						
70–80	0.550	0.372	2.185	0.139	1.734	0.836–3.596
80 and above	−0.051	0.431	0.014	0.905	0.950	0.408–2.212
Marital status (ref: unmarried)						
Married	−3.165	2.992	1.119	0.290	0.042	0.000–14.875
Other	−2.998	2.997	1.001	0.317	0.050	0.000–17.751
Monthly income (ref: <3,000)						
3,000–5,000	0.312	0.332	0.881	0.348	1.366	0.713–2.617
5,000–7,000	1.377	0.728	3.585	0.058	3.965	0.953–16.499
≥7,000	1.401	1.368	1.048	0.306	4.058	0.278–59.305
Education level (ref: junior high school or below)						
High school (vocational/technical secondary school)	−0.029	0.460	0.004	0.949	0.971	0.395–2.391
Other	0.164	0.769	0.045	0.831	1.178	0.261–5.317
Occupation (ref: government/public institution)						
Professional and technical personnel	1.168	0.792	2.173	0.140	3.215	0.680–15.188
Workers	0.107	0.804	0.018	0.894	1.113	0.230–5.384
Farmers	0.621	0.800	0.603	0.438	1.861	0.388–8.929
Other	0.735	0.856	0.737	0.391	2.085	0.389–11.171
Professional and technical personnel	−0.599	1.042	0.330	0.565	0.549	0.071–4.235

## 4 Discussion

The results of this study showed that the prevalence of influenza vaccine hesitancy among older adults in Shihezi City was 54.5%. Multivariable regression analysis demonstrated that vaccine hesitancy was affected by multiple dimensional factors. Complacency, perceptions of vaccine efficacy and safety, and knowledge levels regarding influenza and influenza vaccines were identified as independent influencing factors.

The prevalence of influenza vaccine hesitancy among older adults in Shihezi City is higher than that reported in previous studies in some regions of China. A national cross-sectional study covering 10 provinces in China reported that 37.18% of older adults were hesitant about influenza vaccination (16). A study conducted among older adults in Guiyang City, Guizhou Province, China, showed an influenza vaccine hesitancy rate of 49.13%, with confidence, risk perception, and social support identified as key influencing factors (26). Furthermore, a study based on a nationally representative sample in France reported an influenza vaccine hesitancy rate of 35% among older adults, which was associated with poor self-rated health and negative perceptions of vaccine risks (27).

This study, based on the 5C model, explored the factors influencing influenza vaccine hesitancy among community-dwelling older adults. Psychological factors play a critical role in vaccine hesitancy. In the multivariable analysis, the Complacency dimension score of the 5C model was positively correlated with vaccine hesitancy. Older adults with higher scores had a higher risk of vaccine hesitancy (OR = 3.204). The complacency dimension reflects individuals’ underestimation of disease threats, which further reduces their perception of the necessity of vaccination. The Confidence dimension (trust in vaccine safety and efficacy) was negatively associated with vaccine hesitancy (OR = 0.827), indicating that higher trust in vaccines corresponds to a lower likelihood of vaccine hesitancy. This finding is consistent with the conclusions of numerous domestic and international studies, which have identified concerns about vaccine safety and efficacy as key contributing factors to influenza vaccine hesitancy (28–30). Secondly, compared with older adults who did not recognize the efficacy and safety of influenza vaccines, those who perceived influenza vaccines as effective or safe presented a lower risk of vaccine hesitancy (OR = 0.048, OR = 0.054), indicating that subjective perceptions of vaccine efficacy and safety are correlated with vaccination decisions. Knowledge level is also an influential factor for vaccine hesitancy. This study found that older adults with moderate and high influenza knowledge scores had a lower risk of vaccine hesitancy than those in the low-score group (OR = 0.095, OR = 0.111). Similarly, the high-score group of influenza vaccine knowledge showed a lower risk of vaccine hesitancy than the low-score group (OR = 0.286). These findings suggest that individuals’ understanding of disease risks and vaccine benefits may shape their attitudes toward vaccines, thereby being associated with vaccine hesitancy (31, 32).

In conclusion, according to the results of multivariable logistic regression analysis, influenza vaccine hesitancy among community-dwelling older adults is associated with complacency, insufficient cognition of vaccine efficacy and safety, and low levels of knowledge regarding influenza and influenza vaccines. These findings suggest that future observational studies and intervention strategies should focus on these modifiable factors. In particular, medical staff should give full play to their potential role in risk communication and health education, so as to effectively reduce influenza vaccine hesitancy and improve vaccination coverage among older adults.

## 5 Limitations

Several limitations of this study should be acknowledged. First, the cross-sectional design only allows us to describe the situation at a single time point and precludes any inferences about temporal sequences or causal relationships among the variables. Second, self-report bias may be present, as all data were collected via face-to-face questionnaires, and participants may have over- or under-reported their vaccination history due to social desirability concerns. Third, selection bias is possible because older adults with cognitive impairment, hearing loss, or language disorders were excluded. Consequently, the sample may be skewed toward relatively healthier older adults with normal cognitive function, which limits the generalizability of our results.

## Data Availability

The original contributions presented in the study are included in the article/supplementary material, further inquiries can be directed to the corresponding author.
